# Metformin Degradation by Advanced Oxidation Processes: Performance, Limitations, and Environmental Concerns

**DOI:** 10.3390/ijms26135925

**Published:** 2025-06-20

**Authors:** Jaime M. Castañeda-Sánchez, Felipe de J. Silerio-Vázquez, Ignacio Villanueva-Fierro, Juan Carlos García-Prieto, Luis A. González-Burciaga, José B. Proal-Nájera

**Affiliations:** 1 CIIDIR-Unidad Durango, Instituto Politécnico Nacional, Calle Sigma 119, Fracc. 20 de Noviembre II, Durango 34220, Durango, Mexico; 2 Centro de Investigación y Desarrollo Tecnológico del Agua, Universidad de Salamanca, Campo Charro s/n, 37080 Salamanca, Spain

**Keywords:** metformin degradation, wastewater treatment, diabetes mellitus, emerging contaminants, advanced oxidation processes, biguanides

## Abstract

This review provides a descriptive analysis of metformin, highlighting its environmental presence and classification as an emerging contaminant. It examines the risks associated with metformin and evaluates advanced oxidation processes (AOPs) for its degradation, including photolysis, photocatalysis, electrolysis, and ozonation. Metformin, a widely used biguanide for type 2 diabetes, is increasingly detected in aquatic environments due to its incomplete metabolism in humans, raising ecological concerns. While certain AOPs, such as ultraviolet (UV) photocatalysis and ozonation, achieve high degradation rates of 99.9% and 100%, respectively, they produce toxic by-products harmful to aquatic systems. Solar photocatalysis, despite a lower degradation rate (74.22%), stands out for operating without artificial energy and generating fewer hazardous by-products. The review identifies gaps in current degradation strategies and underscores the need for clean, sustainable methods. Future research directions include advancing biological and photocatalytic technologies to improve AOPs’ efficiency while minimizing environmental risks.

## 1. Introduction

Emerging contaminants (ECs) are chemical substances that are not included in routine environmental monitoring, and therefore are not regulated or registered by discharge regulatory authorities [1,2], mainly due to the lack of information on the effects produced by chronic exposure [3], so they could be under scrutiny for future legislation [4]. ECs, also known as contaminants of emerging concern or microcontaminants, are synthetic or natural organic chemicals that are released into the environment and can create adverse effects on the natural environment and human health [3,5,6]. According to the NORMAN database, at least 700 substances are classified as ECs, grouped into 20 different classes identified in aquatic environments across Europe [3,6], among which the most prominent are pesticides, pharmaceuticals, personal care products, disinfection by-products [7], perfluorinated compounds, gasoline additives, flame retardants, nano-compounds, endocrine disruptors and some inorganic compounds such as heavy metals [8].

At the same time, ECs can be categorized into three broad groups. The first group comprises compounds that have recently been introduced into the environment. The second group comprises those contaminants that have been detected recently, but have been part of the environment for a long period of time. Finally, there are compounds that are known and have been detected for a considerable time, with their adverse effects on humans and/or ecosystems being recognized [9].

More than 3000 substances are used as medicines, including erectile dysfunction drugs, analgesics, antibiotics, antidiabetics, beta blockers, antidepressants, X-ray contrast agents, contraceptives, lipid regulators, antiseptics, antiepileptics, hormones, anti-inflammatories, chemotherapy drugs, statins, etc. [10,11,12,13,14,15,16]. Some of these compounds are consumed and produced each year in the order of thousands of tons per year [15,17]. Drugs are compounds that have been designed for specific metabolic and molecular pathways for the prevention of diseases and the diagnosis of them, as well as for the treatment of and change in a specific condition [15,18].

The above can be extended to veterinary medicine and can be applied to illicit drugs [17]. In the last 20 years, pharmaceutical compounds have emerged as potential ECs and have attracted attention due to their relative stability [12,19]. Pharmaceutical compounds are bioactive, recalcitrant and bioaccumulative due to their diverse chemical structure and nature that make them resistant to natural biodegradation making them bioaccumulative and bioactive [7,20]. Depending on their use, dose and degradation route within living organisms, they can cause endocrine disruption and reproductive abnormalities, and may promote bacterial resistance and ecotoxicity even at low concentrations [21].

In developing communities, adequate sanitation facilities are lacking or scarce; thus pharmaceutical compounds enter the environment through several sources [13,22]. Among these sources, the most important are hospital waste, pharmaceutical industry, improper disposal of medicines, domestic areas, aquaculture and farms [21,22,23] as a result of uncontrolled urbanization, industrial development, agriculture, transportation and other anthropogenic activities [1,3].

The pharmaceutical industry is noted for consuming 22% of the total fresh water, generating a large amount of wastewater that is not fully treated or does not undergo any treatment [18]. However, 90% of pharmaceutical active compounds enter domestic wastewater through excretion [12], as metabolites or the unchanged compound, due to the lack of complete metabolization of these compounds by humans and animals [21,24].

These contaminants reach wastewater treatment plants (WWTPs), which are inefficient in degrading or removing them since they are usually designed to mainly degrade macronutrients, and reduce organic matter, nitrogen and phosphorus [12,24,25,26], which causes the presence of pharmaceuticals in fresh surface water and groundwater [24] due to a constant supply of drugs in aquatic matrices [21]. The existence of these contaminants in drinking water raises great concern for the risk to health and aquatic ecosystems due to prolonged exposure. Wastewater and drinking water treatment processes are usually not specifically designed to remove pharmaceuticals [2]. This is particularly worrying because the biological response produced by drugs to target organisms produces the same response to non-target entities triggering a chronic harmful effect [12].

This paper reviews global information on the presence of metformin (MET) in water matrices and examines the effectiveness of different AOPs applied to this contaminant, along with the degradation by-products. This work focuses on the individual processes of photocatalysis, photolysis, electrolysis, ozonation, Fenton-type reactions and any combination of these, analyzing the most recent advances in MET degradation. The objective of this review is to highlight the potential of AOP technologies for water treatment applications and contribute to the growing body of knowledge on the management of pharmaceutical contaminants in aquatic environments.

A thorough literature search was conducted to identify publications that met the selection criteria established by the authors, which focused on studies examining MET degradation by AOPs. To this end, a comprehensive exploration of pertinent scientific databases, such as Google Scholar, Scopus and ResearchGate, was conducted to identify relevant studies published between 2014 and 2024. To supplement the findings, updated population data were obtained from the International Diabetes Federation (IDF), to contextualize MET consumption trends and potential environmental impact. The selection of articles was conducted using a combination of keywords, including metformin, degradation, contaminant removal, photocatalysis, AOPs, oxidation and by-products.

This review article methodically compiles and organizes these studies, describing various degradation processes while analyzing their potential and limitations. Additionally, it provides a comprehensive overview of developments in this field, offering valuable insights for researchers, policymakers, organizations and professionals interested in its application and regulation. By synthesizing the latest findings, this review aims to support ongoing research and inform regulatory decisions related to metformin degradation and water treatment technologies.

## 2. Diabetes Mellitus

### 2.1. Diabetes Mellitus Classification

Diabetes is a metabolic disorder characterized by deficiency in insulin production, insulin action or both, leading to hyperglycemia in the absence of adequate treatment. Metabolic alterations involving the assimilation of carbohydrates, fats and proteins are a consequence of the disease, along with long-term complications such as retinopathy, nephropathy and neuropathy. Diabetics are at risk of other diabetes-related conditions such as cardiovascular and cerebrovascular diseases, obesity, eye conditions, erectile dysfunction, tuberculosis and non-alcoholic fatty liver disease [27]. Most cases of diabetes can be classified into two main groups: type 1 diabetes and type 2 diabetes; however, there are some cases that are more difficult to classify included in another group: other specific types. Gestational diabetes is another classification characterized by glucose intolerance with onset or first recognition during pregnancy [28].

Type 1 diabetes is characterized by a total insulin deficiency in which those affected develop ketoacidosis, which can lead to coma that can lead to death if insulin is not administered. The disease commonly occurs in childhood and adolescence, but is not specific to this demographic. Type 1 diabetes can be separated into type A1 and type A2, accounting for 5% to 10% of total diabetes cases [29]. Type 2 diabetes, previously called “non-insulin-dependent diabetes” or “adult-onset diabetes,” accounts for 90–95% of individuals with the condition. This form encompasses individuals who have a relative, rather than absolute, deficiency of insulin and have peripheral insulin resistance. At least initially, and often for life, these individuals may not require insulin treatment to survive [30].

Pregnancy-related hyperglycemia increases the risk of poor outcomes for the mother, fetus and newborn. This risk is present whether hyperglycemia takes the form of type 2 diabetes diagnosed before or during pregnancy. Newborns of mothers with gestational diabetes are at increased risk of developing diabetes in adulthood. The increased incidence of pregnancy-related complications such as preterm birth, large-for-gestational-age births, macrosomia (birth weight > 4.5 kg), cesarean delivery and pre-eclampsia is mainly due to hyperglycemia during pregnancy, which results in larger neonates [31].

Types of diabetes mellitus of various known etiologies are grouped into the classification called, as mentioned above, “other specific types”. This type of diabetes was previously called “Maturity Onset Diabetes of the Young” (MODY) or maturity onset diabetes in youth. This group includes people with genetic defects of beta cell function or with defects of insulin action; people with diseases of the exocrine pancreas, such as pancreatitis or cystic fibrosis; people with dysfunction associated with other endocrinopathies; and people with pancreatic dysfunction caused by drugs, chemicals or infections, and represent less than 10% of cases of diabetes mellitus [32].

### 2.2. Diabetes Mellitus: Cases and Deaths

In 2021, Sun et al. [33] analyzed 219 sources of information from 144 countries to estimate the number of diabetes cases worldwide, yielding a result of 536.6 million people suffering from diabetes in adults between 20 and 79 years of age across 215 countries and territories. The information was used to project the number of cases of this disease for the year 2045, with 783.2 million suffering from diabetes. According to the IDF, in 2021, the Middle East and North Africa region has the highest prevalence of diabetes in people aged 20 to 79 years, at 18.1%. This figure is expected to increase in the future, reaching 20.4% in 2045. On the other hand, the African region has the lowest prevalence of diabetes comparatively, at 5.3% in 2021 (Table 1). This is partly due to low levels of urbanization and a lower prevalence of overweight and obesity. However, the prevalence in this region is expected to increase slightly, reaching 5.2% in 2045. This increase is expected to be higher in other IDF regions. It is important to note that these estimates might underestimate the true prevalence, given rapid urbanization and lifestyle and environmental changes in the African region [34].

About 6.7 million people aged 20–79 years died from diabetes or its complications in 2021, corresponding to 12.2% of all deaths in this age range. The IDF region with the highest number of estimated diabetes-related deaths in the same age range is the Western Pacific with just under 2.3 million. The next region with the highest number of estimated deaths is Europe with just over 1.1 million, with the widest difference between IDF regions [34]. Table 2 shows the estimated diabetes-related deaths in 2011 and 2021 among people 20–79 years of age, as well as cases of type 1 diabetes in children and adolescents up to 19 years of age.

### 2.3. Treatment for Diabetes Mellitus

**Figure 1:**
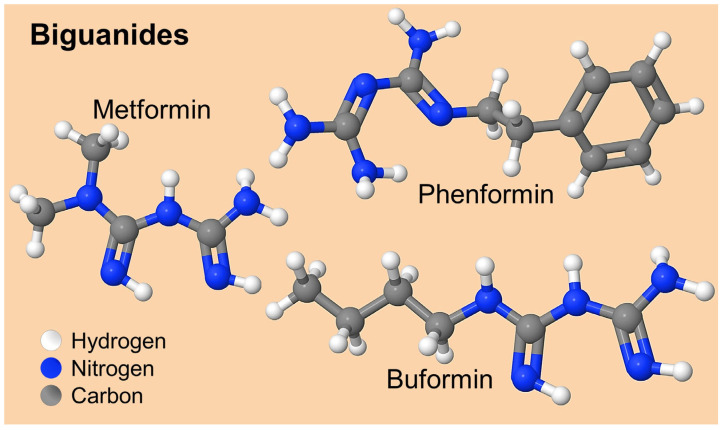
Chemical structure of biguanides for the treatment of diabetes mellitus.

Some of the most widely used treatments for diabetes mellitus are a class of hypoglycemic agents called biguanides. Notable biguanides include MET, phenformin (PHF) and buformin (BUF), each with distinct characteristics and safety profiles (Figure 1).

MET, introduced in 1957, has established itself as the first-line treatment for type 2 diabetes mellitus, due to its ability to reduce hepatic glucose production and improve insulin sensitivity in peripheral tissues, thereby lowering blood glucose levels without causing hypoglycemia or inducing weight gain, being particularly suitable for overweight patients; in addition, it has benefits for the lipid profile by reducing triglycerides and low-density lipoprotein (LDL) cholesterol [35].

PHF, although effective in lowering glucose, has fallen into disuse because of its association with an increased risk of lactic acidosis, a serious side effect that has limited its use and led to its discontinuation in favor of MET [36]. On the other hand, BUF, introduced in 1958, also demonstrated efficacy in controlling hyperglycemia, but its clinical application has been restricted due to the lack of sufficient data on its safety and the availability of safer alternatives, which has reduced its use compared to MET [37]. The most important physicochemical properties of drugs for treating diabetes mellitus from the biguanide group are shown in Table 3.

There are also other alternative drugs to biguanides, such as sitagliptin and saxagliptin, which act by different mechanisms to improve glycemic control in patients with type 2 diabetes. Dipeptidyl peptidase (DPP-4) inhibitors prolong the action of incretins, improving insulin secretion without increasing the risk of hypoglycemia. Similarly, glucagon-like peptide-1 (GLP-1) hormone analogues, such as liraglutide and dulaglutide, promote insulin secretion and slow gastric emptying, which may also help reduce body weight. On the other hand, sodium–glucose co-transporter 2 (SGLT2) protein inhibitors, such as canagliflozin and empagliflozin, increase urinary glucose excretion and offer additional cardiovascular and renal benefits [41].

### 2.4. Occurrence of Metformin in Water Matrices

In 2017, more than 100 million prescriptions for MET were made in England and the United States alone [42] and it is currently used by more than 200 million patients around the world as monotherapy or in combination with sulfonylureas or DPP-4 inhibitors for the treatment of type 2 diabetes mellitus [43]. According to the CHEMANALYST website, the MET market size was 75,000 tons in 2023 worldwide and a growth of 4.5% is projected towards 2034 [44]. This, coupled with an incomplete metabolism in the human body, has led to its detection in aquatic matrices, which has raised concerns about its ecological impact on non-target organisms. The evaluation of the environmental exposure and effects of this drug is crucial to assess the associated risks and ensure the sustainability of aquatic ecosystems [45].

**Figure 2:**
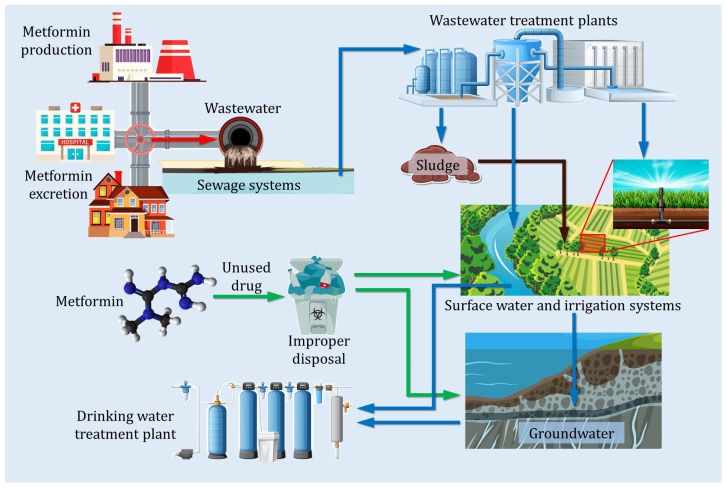
Proposed route of metformin through different water matrices during its lifetime (green arrows indicate direct release via improper disposal to surface waters and irrigation systems; blue arrows show pathways through wastewater and drinking-water treatment plants to surface water and groundwater; brown arrow denotes return of MET in sludge applied to soil). Modified from Briones et al., 2016 [47].

MET contamination has been identified in several environmental sectors. In WWTPs, this compound, as well as its by-products, has been detected in both influents and effluents, with levels ranging from 3200 to 129,000 ng/L in different facilities [46], indicating that these plants have inefficient processes to completely degrade this pharmaceutical compound. It has also been found in surface water bodies, such as rivers, lakes and streams, and its presence has even been reported in drinking water, highlighting the potential risk of human exposure to this contaminant. In addition, its presence has been reported in groundwater samples, possibly due to infiltration, leachates or direct application of wastewater. Finally, MET residues have also been identified in hospital effluents, suggesting that hospitals are significant sources of pharmaceutical contamination, derived from both patient excretion and inadequate disposal practices [42]. Figure 2 shows the path of MET waste from its manufacture in industry, hospital effluents and domestic sources to its final destination in the environment.

In the European Union (EU), MET and its persistent metabolite guanylurea were added to the Union-wide Watch List for emerging substances (Article 8b of Directive 2008/105/EC) [48] by Commission Implementing Decision (EU) 2025/439 [49]. Their inclusion reflects widespread detection in surface waters and the potential for additive ecotoxicological effects.

Placing MET and guanylurea on the Watch List compels member states to generate standardized, high-quality monitoring data using harmonized analytical methods, which is critical for assessing environmental concentrations, risks to aquatic organisms and human-health implications given MET’s rising consumption and low removal in conventional treatment plants. This monitoring underpins key EU policies, including the Strategic Approach to Pharmaceuticals in the Environment (COM/2019/128 final) [50] and the One Health Action Plan against Antimicrobial Resistance (COM/2017/339 final) [51], and will provide the evidence base needed to strengthen mitigation measures. Ultimately, more robust data on MET’s fate and transport will guide the integration of advanced oxidation and other novel treatment technologies into regulatory frameworks.

Building on these regulatory drivers, this review critically evaluates the performance, limitations and scale-up potential of advanced oxidation processes (AOPs) for metformin degradation in wastewater treatment.

## 3. Metformin Degradation by Advanced Oxidation Processes

MET waste contains recalcitrant compounds, which makes it difficult to degrade, and there are various methods capable of reducing or eliminating them. These techniques can be through biological processes, such as the microbial activity of specific bacteria or organisms that gradually decompose the compound, as well as physical or chemical processes, such as hydrolysis, photolysis or reactions with natural minerals. On the other hand, there are AOPs, degradation technologies that are characterized by generating highly reactive species, such as hydroxyl radicals (^•^OH), useful for oxidizing and decomposing persistent and recalcitrant organic compounds in water systems, such as microcontaminants, pharmaceuticals and personal care products, obtaining high removal efficiencies, even for low concentrations of contaminants. These processes include photocatalysis, ozonation, UV irradiation, and the use of oxidizing agents such as hydrogen peroxide (H_2_O_2_) and persulfates [52].

Over the past few years, research into AOPs has been growing, generating advances in efficiencies, method optimization and applicability in different systems. These processes can be applied to various water treatment scenarios, including drinking water treatment, wastewater treatment and groundwater remediation, as well as to different levels of contamination. Another positive aspect is that they do not require the addition of chemicals, such as coagulants or flocculants, for the removal of contaminants, which reduces the amount of chemicals used in water treatment and minimizes sludge production. They are also capable of effectively degrading contaminant transformation by-products that are formed during the degradation of MET [53]. Experiments on the degradation of MET by different AOPs are shown in Table 4.

**Figure 3:**
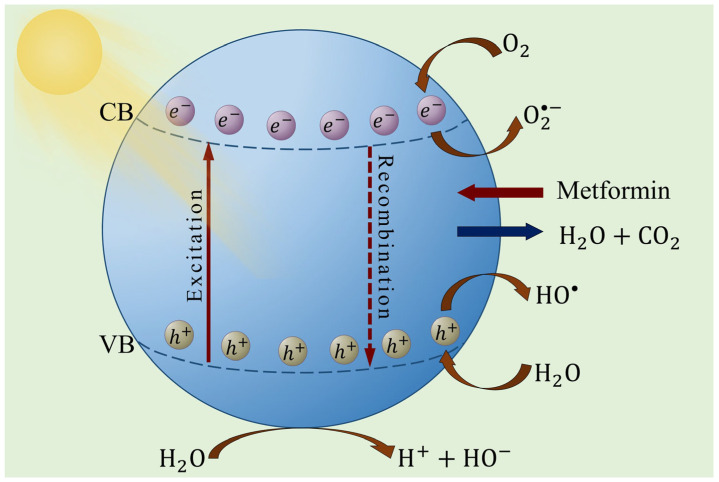
Illustration of the photocatalytic mechanism showing the generation of the electron–hole pair responsible for the redox reactions that degrade organic contaminants.

Photocatalysis leverages the interaction between light and semiconductor materials to drive chemical reactions. When exposed to photons with energy equal to or greater than the bandgap of the material, electrons are excited from the valence band (VB) to the conduction band (CB), leaving behind holes [71,72]. These electron–hole pairs can participate in redox reactions, producing reactive oxygen species such as hydroxyl radicals (Figure 3), which are crucial for breaking down organic contaminants and other environmental contaminants [73]. The efficiency of this process is influenced by the bandgap, electron–hole recombination rate and the surface characteristics of the catalyst [74]. Recent advancements in material synthesis, including metal doping, heterostructure formation and nanoscale engineering, aim to enhance light absorption and electron mobility, enabling greater degradation efficiencies under visible and UV light [71,73].

In recent years, the application of AOPs for MET degradation has increasingly focused on photocatalysis using TiO_2_, doped TiO_2_ and more complex materials. Nezar and Laoufi [57] studied the photocatalytic degradation of MET using various TiO_2_ photocatalysts (P25, DT51, PC105, T42, EL10 and PC500) under UV radiation. TiO_2_-P25 demonstrated the highest efficiency, achieving 62% degradation in 240 min, followed by DT51 at 53%. Both catalysts had similar crystallite sizes (~26 nm) and surface areas (~55 m^2^/g), but TiO_2_-P25 contained 80% anatase and 20% rutile, while DT51 was 100% anatase [57]. Although anatase is generally more photocatalytically active than rutile [75,76], the mixed phase of TiO_2_-P25 may explain its 9% higher efficiency [77]. For solar-driven photocatalysis, a laboratory-scale reactor with TiO_2_-P25 (0.1 g/L) achieved 83% degradation, outperforming photolysis (67%) and UV lamp-driven processes (62%). The addition of electron acceptors, particularly sodium persulfate (Na_2_S_2_O_8_;), enhanced degradation to 99% at 1 g/L after 210 min [57].

Building on these findings, Cheshme et al. [66] synthesized a novel nanostructured hybrid catalyst, Fe_3_O_4_@rGO@ZnO/Ag-NPs (FGZAg), for MET degradation under UV-A and visible light. Among various compositions, FG_10_ZAg_8_ achieved complete MET degradation and 60.9% mineralization in 60 min. The incorporation of Ag-NPs and reduced graphene oxide (rGO) improved electron–hole separation and visible light absorption, making this catalyst effective for pharmaceutical contaminant removal [78,79,80].

Further exploring catalyst optimization, Carbuloni et al. [55] compared six degradation processes, including photolysis and photocatalysis with TiO_2_-P25, TiO_2_ sol-gel, ZrO_2_ sol–gel and TiO_2_/ZrO_2_ composites. TiO_2_/ZrO_2_ (95%/5%) and TiO_2_ sol–gel showed the highest kinetic constants (0.0099 min^−1^ and 0.0081 min^−1^, respectively). However, ZrO_2_-based catalysts suffered from high electron–hole recombination and a broad bandgap (5.0 eV), limiting their efficiency [81].

The sol–gel method has also been used to synthesize efficient photocatalysts. Nematolahi et al. [62] synthesized Fe^3+^/TiO_2_ via this method and evaluated the effects of pH, catalyst dose and initial MET concentration. Degradation exceeded 85% across pH 3–11, with an optimal performance at pH 11 due to electrostatic attraction between the negatively charged catalyst surface and cationic MET (pKa ~2.8 and ~11.5) [82,83]. Alkaline conditions also enhanced ^•^OH generation, crucial for degradation [46]. Catalyst loading above 35 mg/L had a minimal impact, while higher MET concentrations reduced efficiency due to surface saturation.

Expanding on catalyst innovation, Iqbal et al. [70] synthesized lanthanum (La)-doped WO_3_ catalysts using *Azadirachta indica*, achieving 93% degradation due to its low bandgap and small particle size (65 nm). Smaller particles enhance surface area and active site availability, improving efficiency [84,85].

Metal oxides have been widely used in photocatalysis using only UV radiation due to their wide band gap, which limits their practical applications due to the high cost of using such light sources [86,87]. Metal doping can reduce the band gap, allowing the use of the visible spectrum of light, but this process can be complex and expensive, which can hinder large-scale applications and commercial viability [87,88].

Some metal-doped catalysts exhibit good photostability over multiple cycles of use, different conditions and extended periods of time. However, the structure of the catalysts can degrade, affecting their long-term performance [89]. The use of certain metals in doping processes can raise environmental and health concerns. The potential toxicity of these materials, especially when used in large quantities, must be carefully managed to avoid adverse effects [86,88].

Kumar et al. [68] explored the effects of different UV regions (UV-A, UV-B and UV-C) on MET degradation. UV-B, with its higher photon emission, proved most effective, achieving >99% degradation in 60 min using a poly(3,4-ethylenedioxythiophene) (PEDOT) catalyst at 0.5 g/L and pH 5.6. Acidic conditions (pH 2.8–5.6) were optimal, aligning with the point of zero charge of PEDOT (3.88–4.55), which favors electrostatic attraction with MET [90,91].

Research is underway to identify alternative co-catalysts to noble metals, which are expensive and in short supply. Materials such as MXenes are being explored as cost-effective alternatives due to their unique properties and potential to improve photocatalytic efficiency [92]. Integrating photocatalysis with other technologies, such as membrane filtration, is being investigated to enhance the overall efficiency and applicability of the process. This hybrid approach has the potential to address some of the current limitations [93].

**Figure 4:**
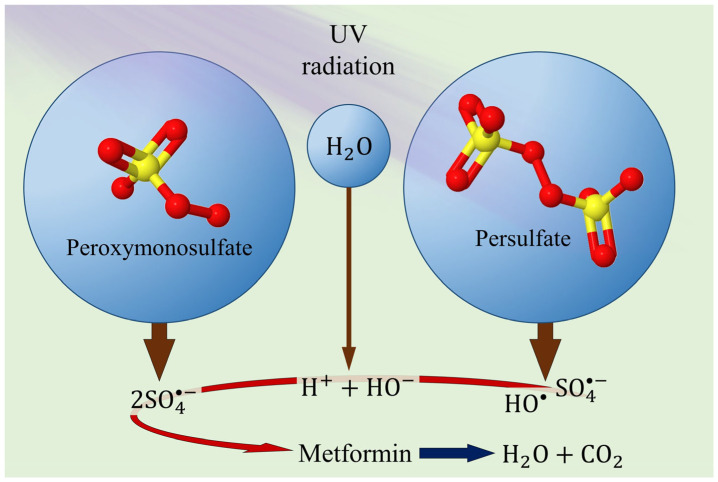
Illustration of the photocatalytic mechanism from peroxymonosulfate and persulfate, responsible for the degradation of organic contaminants. Modified from Xiao et al. [98] and Milh et al. [99].

Sulfate and sulfite are increasingly used in photocatalysis for water decontamination due to their ability to enhance the degradation of organic contaminants. These processes leverage the generation of reactive radicals to break down contaminants effectively (Figure 4). Sulfite has been shown to significantly enhance the photocatalytic degradation of organic contaminants. This can be attributed to the formation of sulfite radicals, which are highly reactive and have been observed to decompose contaminants, which occurs through a reaction with holes or ^•^OH, and this mechanism has been found to be effective in various photocatalysts, such as TiO_2_ [94,95].

Sulfate radicals (SO_4_^•−^) are potent oxidants utilized in AOPs for water treatment. These radicals can be generated by activating sulfate compounds, such as peroxymonosulfate or persulfate [96,97]. In situ photochemical activation of sulfate to produce SO_4_^•−^ is a promising method. This method eliminates the need for additional chemicals, making it more sustainable and cost-effective [96].

Karimian et al. [63] further advanced MET degradation by combining VUV/Fe/PMS, achieving 99.4% degradation and 70.3% mineralization in 60 min at pH 6.3. The synergy between Fe(II) and PMS significantly enhanced performance, offering a promising approach for nitrogen-containing pharmaceuticals. Similarly, Parra-Marfil et al. [61] used UV-activated persulfate, achieving 26.1–87.3% degradation, with higher efficiency at low MET and high oxidant concentrations. However, mineralization was incomplete (15.1–64%), indicating by-product formation, which remains a challenge for practical applications.

Recent investigations, such as the Gu et al. [69] use of UV-irradiated sulfite, achieved 96.54% degradation in half an hour under optimal conditions (10 mM sulfite, pH 8–9). Meanwhile, Tominaga et al. [59] employed electron beam irradiation (EBI), achieving almost complete removal and 65.41% mineralization at 5 kGy, though by-products increased toxicity.

AOPs with sulfite and sulfate demonstrate considerable potential for the degradation of MET. However, substantial technical and economic challenges must be addressed. The existing literature identifies several limitations regarding activation, efficiency and by-product control.

Sulfite-based AOPs require high sulfate ion concentrations in UV processes; however, the generation of hydrated electrons is limited by their reaction with other species, thereby diminishing degradation efficiency. Moreover, using activators like metal ions and powdered catalysts complicates their recovery, potentially causing secondary contamination, while the resulting by-products may be more toxic in complex matrices [100].

In sulfate-based processes, high energy and reagent consumption elevates operating costs, limiting industrial viability relative to biological methods. Additionally, the formation of persistent and potentially more toxic by-products confines these studies primarily to the laboratory scale [101]. Although peroxymonosulfate is easy to activate, maintaining sulfate ion levels below the regulatory threshold to protect aquatic life is challenging, and its high cost further impedes practical application [102].

Peroxymonosulfate and persulfate also exhibit high reactivity with organic and inorganic compounds such as humic acids, metals and chloride ions, which reduces efficiency by promoting the formation of reactive intermediates; furthermore, the released sulfate may induce the production of sulfuric acid [103].

Ozonation has shown promising results for MET degradation due to its rapid oxidation potential and short contact time requirements. Fujinami [64] reported complete removal of MET within 10 min using an ozone dosage of 100 mg/h under UV irradiation. This high efficiency is attributed to both direct oxidation by ozone and the generation of reactive radicals, such as hydroxyl radicals (^•^OH), through ozone decomposition. Furthermore, coupling ozone with UV and H_2_O_2_ has been shown to significantly enhance degradation performance, underscoring the potential of hybrid AOP strategies.

However, despite its high degradation efficiency, ozonation achieved only moderate mineralization (~20%) [58], indicating the accumulation of partially oxidized intermediates, which may pose ecological risks. These limitations, along with the high operational costs associated with ozone generation and system maintenance, challenge its practicality compared to photocatalysis, which often surpasses 90% degradation using inexpensive and solar-active catalysts like TiO_2_. Therefore, while ozonation remains attractive for rapid contaminant removal, its environmental and economic drawbacks must be carefully weighed, particularly in large-scale applications.

The optimization of operational parameters in MET degradation through AOPs requires careful consideration of multiple interconnected variables that significantly influence treatment efficiency. Solution pH represents a critical parameter affecting both radical generation mechanisms and catalyst–contaminant interactions, with acidic conditions typically promoting positive catalyst surface charges that enhance interactions with negatively charged contaminants, while alkaline conditions (pH 9–12) may favor certain degradation pathways despite potential ^•^OH scavenging by carbonate and bicarbonate ions [104,105].

Catalyst and oxidant dosage optimization involves balancing reactive oxygen species generation with potential adverse effects. Optimal catalyst concentrations ensure maximum active sites for light absorption and radical generation, though excessive dosages can lead to light blocking, reduced surface area effectiveness and secondary contamination concerns [105,106]. Similarly, H_2_O_2_ concentration must be carefully controlled, as, while higher concentrations enhance ^•^OH formation, excessive amounts may result in radical scavenging and unwanted by-product formation [107,108].

Temperature and reaction time optimization directly influence degradation kinetics, with elevated temperatures generally promoting enhanced reaction rates while requiring consideration of energy costs and potential catalyst deactivation. The degradation process typically follows pseudo-first-order kinetics, emphasizing the importance of maintaining optimal conditions throughout the treatment duration [106]. Reactor design considerations, including geometry, operational mode and irradiation intensity, significantly impact mass transfer characteristics and overall process efficiency. Additionally, competing substances in real wastewater matrices can interfere with metformin degradation by scavenging reactive species, necessitating parameter adjustments to account for matrix effects and ensure effective pharmaceutical removal [107].

The systematic integration and fine-tuning of these operational parameters not only enhance metformin removal efficiency but also plays a crucial role in controlling the formation of transformation products, which is essential for the safe and practical implementation of AOP-based pharmaceutical wastewater treatment systems. This comprehensive understanding of parameter optimization provides a fundamental framework for examining the nature, toxicity and environmental implications of the degradation intermediates and by-products generated during metformin oxidation processes.

## 4. Metformin Degradation By-Products

The degradation of MET leads to the possible generation of by-products, which are chemical compounds produced when it decomposes in the environment, either by natural processes, such as exposure to sunlight, or induced processes, such as AOPs. The identification and study of these by-products is essential because some may be persistent or toxic to aquatic organisms and human health, and also allows us to better assess the environmental risks associated with the widespread use of the drug and optimize the treatment of contaminant removal in wastewater [109]. Table 5 shows MET by-products generated during different AOPs.

The information presented in Table 5 highlights the formation of various by-products from the degradation of MET using different AOPs, each with distinct degradation mechanisms based on the reactions involved.

Parra-Marfil et al. [61] proposed a general mechanism for MET degradation by sulfate radicals, identifying six primary by-products: N-cyanoguanidine, N,N-dimethyl-urea (DMU), N,N-dimethyl-cyanamide, N,N-dimethyl-formamide (DMF), glycolonitrile and guanidine. Based on HPLC-EIS-MS analyses, they suggested three degradation pathways. In contrast, Gu et al. [69], using the UV/sulfite system, detected three intermediate compounds 4,2,1-AIMT, methylbiguanide, and 2,4-AMT. Unlike Parra-Marfil et al. [61], they did not propose a detailed degradation pathway.

Liao et al. [111] investigated MET degradation using O_3_ and O_3_/tert-butyl alcohol (t-BA), proposing four degradation pathways leading to simpler compounds such as N-nitrosodimethylamine (NDMA) and cyanamide. The use of t-BA resulted in only one additional by-product, 1,2-dihydro-3H-1,2,4-triazol-3-imine, which was incorporated into the proposed pathway. Similarly, Badran et al. [113], using the oxy-cracking method, identified 11 compounds and proposed four degradation pathways, including the intermediate ions involved in their formation.

**Figure 5:**
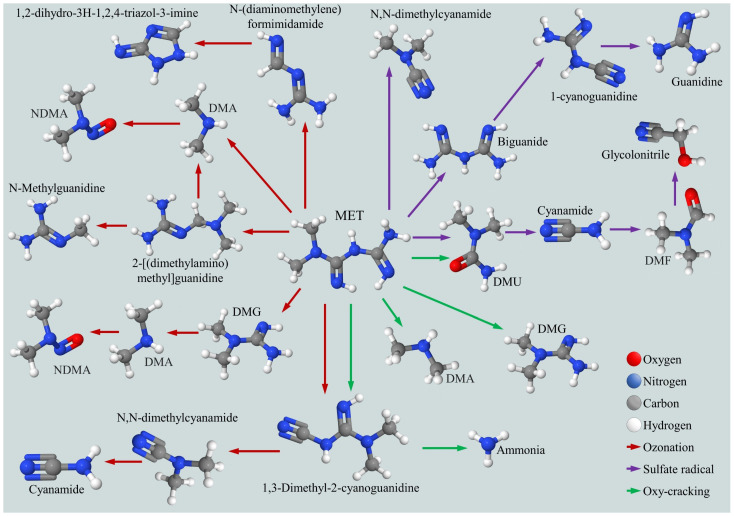
Mechanism of by-product generation from metformin degradation by ozonation (red), advanced oxidation using sulfate radical (purple) and oxy-cracking (green).

Figure 5 combines the degradation pathways and by-product formation from three processes: ozonation, sulfate radical-based AOPs and oxy-cracking. The works of Parra-Marfil et al. [61], Liao et al. [111] and Badran et al. [113] were selected due to their comprehensive and recent proposals of degradation routes leading to simpler molecules compared to MET. Although the authors detected and proposed intermediate radicals, these were excluded from the figure for simplification and space considerations, focusing only on the final by-products.

According to Table 5, TiO_2_-based photocatalysis [58,110] tends to generate nitrogenous by-products such as NH_4_^+^-N, NO_2_^−^-N and NO_3_^−^-N, which pose environmental risks like eutrophication and toxicity in aquatic ecosystems. These limitations can be mitigated through catalyst modifications, such as metal or compound doping, which enhance solar light efficiency and reduce secondary by-product formation. In contrast, PEDOT-based systems [68] excel in utilizing visible light, offering high efficiency for MET degradation. However, they produce by-products like methylbiguanide and guanidine derivatives, which require further evaluation to minimize environmental risks.

Similarly, ozonation [111] and sulfate radical-based processes [69] are highly efficient for contaminant degradation. However, ozonation can generate hazardous compounds like NDMA, while sulfate radicals produce persistent nitrogenous by-products. Both techniques strike a balance between efficiency and risks, emphasizing the need to optimize operational conditions to reduce by-product accumulation. Gamma radiolysis [112], although efficient, faces limitations due to high costs and radiation regulations, restricting its use to specific applications.

In a broader context, degradation by-products from AOPs are present to varying degrees in emerging contaminants. Gamma radiolysis, for instance, generates fewer hazardous by-products compared to other AOPs when degrading contaminants beyond MET [112,114]. Photocatalysis remains one of the most effective techniques for pharmaceutical removal, with studies showing that catalysts like CuO-TiO_2_ and Ag/ZnO achieve over 90% degradation efficiency for compounds such as acetaminophen and industrial dyes [115]. According to Adeyemi et al. [116], heterogeneous photocatalysis under UV or visible light irradiation yields the best results for mineralizing pharmaceuticals in wastewater, including tetracycline, metronidazole, carbamazepine and sulfamethazine.

Furthermore, AOPs have shown promising results in degrading complex organic compounds, such as dyes, pharmaceuticals and aromatic compounds like BTEX [117]. However, the degradation of low-concentration antibiotics remains challenging, though the combination of AOPs has demonstrated potential in addressing this issue.

## 5. Future Directions

The implementation of AOPs for the degradation of microcontaminants in water requires an engineering-driven strategy to deliver treatment systems that are simultaneously scalable, cost-effective and energy-efficient. Future research should focus on closing the gap between promising laboratory-scale results and practical application, paying special attention to the specific challenges identified in this review, such as toxic by-product formation and operational viability.

A fundamental step is the transition from studies focused on reaction chemistry to process engineering. While the development of novel catalysts—such as carbon-based materials, bio-inspired catalysts and heterojunction photocatalysts—is promising for mitigating deactivation and metal leaching, their true potential can only be realized through optimized reactor design. Research should include comparative studies that systematically evaluate how different reactor configurations directly impact metformin degradation efficiency and, crucially, its by-product formation pathway. The development of robust techniques for immobilizing advanced catalysts onto stable substrates is equally vital to facilitate their use in continuous-flow reactors, addressing the challenges of recovery and preventing secondary contamination. Innovations such as immobilized catalyst beds, modular reactor modules and tailored light-delivery systems have the potential to substantially improve performance by optimizing mass transfer and light distribution.

Given that a central conclusion of this review is the formation of potentially toxic by-products even with high degradation rates, future research must prioritize environmental safety. It is necessary to move beyond the simple identification of compounds to assess the aggregate ecotoxicity of the by-product mixture generated by each AOP, a more representative approach to real environmental conditions. Furthermore, hybrid or sequential AOP systems designed specifically to achieve complete mineralization should be investigated, such as combining an initial ozonation process to rapidly break down metformin with a subsequent solar photocatalysis step to degrade persistent intermediates like guanylurea.

It is imperative to validate these technologies in complex water matrices to assess their real-world applicability. Studies should be conducted to systematically quantify the wastewater matrix effect, evaluating the performance of AOPs in real municipal and hospital effluents to determine how background organic matter and inorganic ions affect efficiency and degradation pathways. Alongside this, long-duration experiments are essential to evaluate catalyst stability and system performance over time. The integration of renewable energy sources, particularly solar irradiation, into these systems is a compelling path to reduce operational costs and improve environmental sustainability.

The incorporation of advanced modeling and real-time monitoring tools can significantly enhance the optimization of AOP performance. Dynamic control systems equipped with sensors and online analytics allow for the precise and adaptive regulation of key operational parameters, ensuring consistent treatment efficiency while minimizing hazardous by-product formation. Finally, comprehensive techno-economic analyses and life cycle assessments are vital to confirm the environmental and financial viability of the engineered systems. Fostering interdisciplinary collaboration among material scientists, process engineers and environmental technologists will be fundamental to positioning AOPs as a sustainable and effective strategy for pharmaceutical removal in water treatment.

## 6. Conclusions

A comprehensive literature review indicates that AOPs hold considerable potential for MET degradation in aqueous matrices, a critical issue given the extensive and growing use of this pharmaceutical agent in the treatment of type 2 diabetes, its status as an emerging contaminant and its widespread presence in aquatic environments. Techniques such as photocatalysis, ozonation and sulfate radical-based AOPs, have achieved degradation efficiencies exceeding 90% under optimized conditions. Other techniques, such as photolysis, γ-radiolysis and oxy-cracking, have also been examined; nevertheless, these methods generally exhibit limited efficiency and viability. For example, TiO_2_-based photocatalytic systems, especially when enhanced by metal doping or combined with carbonaceous materials, improve light absorption and electron–hole splitting, thus facilitating the effective oxidation of MET.

However, several limitations hinder the transition from laboratory studies to practical applications. Notably, the formation of toxic by-products, including nitrogenous compounds (NDMA, ammonium, nitrite and nitrate) and intermediates (e.g., methylbiguanide and guanidine derivatives), raises significant environmental and health concerns. In addition, although solar-driven photocatalysis is energy efficient and produces fewer hazardous by-products, it typically yields lower degradation rates than systems employing artificial energy sources. Economic and technical challenges such as high catalyst costs, substantial energy consumption, and complex reactor configurations may hinder scalability. Moreover, optimizing key operational parameters (e.g., pH, catalyst loading and oxidant concentration) remains a complex and demanding task, particularly when dealing with issues such as catalyst deactivation and metal leaching.

Overall, while AOPs show considerable promise in mitigating pharmaceutical contamination, a cautious and multidisciplinary approach is essential to balance degradation efficiency with by-product toxicity.

## Figures and Tables

**Table 1:** Prevalence of diabetes in adults (20–79 years) in IDF regions in 2021 and 2045, ranked by age-adjusted comparative prevalence of diabetes in 2021.

Rank	IDF ^1^ Region	2021			2045		
		People with DM ^2^ (Millions)	DM Prevalence	Comparative DM Prevalence	People with DM (Millions)	DM Prevalence	Comparative DM Prevalence
	World	536.6	10.5	9.8	783.2	12.2	11.2
1	MENA ^3^	72.7	16.2	18.1	135.7	19.3	20.4
2	NAC ^4^	50.5	14.0	11.9	62.8	15.2	14.2
3	SEA ^5^	90.2	8.7	10.0	151.5	11.3	11.3
4	WP ^6^	205.6	11.9	9.9	260.2	14.4	11.5
5	SCA ^7^	32.5	9.5	8.2	48.9	11.9	9.8
6	EUR ^8^	61.4	9.2	7.0	69.2	10.4	8.7
7	AFR ^9^	23.6	4.5	5.3	54.9	5.2	5.6

^1^ International Diabetes Federation; ^2^ Diabetes mellitus; ^3^ Middle East and North Africa; ^4^ North America and Caribbean; ^5^ South-East Asia; ^6^ Western Pacific; ^7^ South and Central America; ^8^ Europe; ^9^ Africa.

**Table 2:** Prevalence of type 1 diabetes in children and adolescents aged 0 to 19 years in 2021. Diabetes-related deaths in adults up to 79 years of age in 2011 and 2021.

Region	DMT1 ^1^ Children and Adolescents 0–19 Years (2021)	Diabetes-Related Deaths in Adults 20–79 Years (2011)	Diabetes-Related Deaths in Adults 20–79 Years (2021)
WP ^2^	2839	1,708,300	2,281,732
EUR ^3^	4977	600,000	1,111,201
NAC ^4^	8371	280,800	930,692
MENA ^5^	9166	276,400	796,362
SEA ^6^	34,930	1,156,000	747,367
AFR ^7^	1240	344,500	416,163
SCA ^8^	6385	227,200	410,206

^1^ Diabetes mellitus type 1; ^2^ Western Pacific; ^3^ Europe; ^4^ North America and Caribbean; ^5^ Middle East and North Africa; ^6^ South-East Asia; ^7^ Africa; ^8^ South and Central America.

**Table 3:** Physicochemical properties of biguanides: metformin, buformin and phenformin.

Compound	Molecular Weight	Chemical Formula	Kow ^1^	pKa ^2^	Solubility in Water	Reference
Metformin	129.16	C_4_H_11_N_5_	−1.43	2.8 11.5	300 mg/mL	[35,38]
Buformin	157.22	C_6_H_15_N_5_	−1.2	11.52 18.1	1.41 mg/mL	[37,39]
Phenformin	205.27	C_10_H_15_N_5_	−1.02	11.97	210 mg/mL	[36,40]

^1^ The octanol–water partition coefficient quantifies a compound’s hydrophilicity versus lipophilicity, ^2^ negative base-10 logarithm of the acid dissociation constant (Ka), reflecting acid strength.

**Table 4:** Degradation of metformin in water matrices by advanced oxidation processes.

Advanced Oxidation Processes	Conditions and Parameters	Removal Rate	Reference
Heterogeneous photocatalysis-UV/titanium dioxide (TiO_2_)	Reaction Time = 30 min [MET]_0_ = 100 mg/L [TiO_2_] = 1000 mg/L	81%	[54]
Photocatalysis-TiO_2_/zirconium dioxide (ZrO_2_)	Reaction Time = 30 min [MET]_0_ = 10 mg/L [TiO_2_] = 1000 mg/L pH = 8	55%	[55]
Photocatalysis	Reaction Time = 150 min [MET]_0_ = 10 mg/L [TiO_2_] = 563 mg/L pH = 7.6	95.2%	[56]
Photocatalysis-TiO_2_/Na_2_S_2_O_8_ electron acceptors	Reaction Time = 180 min [MET]_0_ = 10 mg/L [TiO_2_] = 1000 mg/L Catalyst = Degussa P25	99%	[57]
Ozonation	Reaction Time = 30 min [MET]_0_ = 10 mg/L pH = 9	60%	[58]
Photolysis-UV/C	Reaction Time = 30 min [MET]_0_ = 10 mg/L	9.20%	
Photocatalysis-TiO_2_/UV-C	Reaction Time = 30 min [MET]_0_ = 10 mg/L [TiO_2_] = 120 mg/L pH = 6	31%	
EBI ^1^ and persulfate addition	[MET]_0_ = 8.2 mg/L Irradiation = 5.0 kGy	99.41%	[59]
Solar-irradiated photocatalysis	Reaction Time = 90 min [MET]_0_ = 10 mg/L [TiO_2_] = 750 mg/L pH = 8	78.33%	[60]
Sulfate radicals (UV/S_2_O_8_^2−^ system)	Reaction Time= 60 min [MET]_0_ = 5 mg/L [K_2_S_2_O_8_] = 2750 µM pH = 6.5	87.3%	[61]
Photocatalysis with Fe^3+^ doped TiO_2_ nanoparticles under UV irradiation	Reaction Time = 150 min [MET]_0_ = 35 mg/L [Fe^3+^/TiO_2_] = 75 mg/L pH = 11	93.8%	[62]
VUV ^2^/Fe/PMS ^3^ process	Reaction Time = 60 min [MET]_0_ = 50 mg/L [Fe] = 0.05 mg/L [PMS] = 20 mg/L pH = 6.3	99%	[63]
Ozonation/UV/H_2_O_2_	Reaction Time = 10 min [MET]_0_ = 4 mg/L UV Intensity = 7 W [H_2_O_2_] = 250 mg/L pH = 7 O_3_ Rate = 100 mg/h	100%	[64]
PMS–gingerbread ingredient-derived carbon-assembled CNT ^4^ foam as the catalyst	Reaction Time = 120 min [MET]_0_ = 7.8 mg/L Catalyst = Gingerbread ingredient-derived carbon-assembled CNT foam Oxidant = PMS	68%	[65]
Heterogeneous photocatalytic oxidation—Fe_3_O_4_@rGO@ZnO/Ag-NPs catalyst	Reaction Time = 60 min [MET]_0_ = 20 mg/L [FGZ_10_Ag_8_] = 1 g/L pH = 5.4	100%	[66]
Galvanostatic electrolysis	Reaction Time = 60 min [Na_2_SO_4_] = 2000 mg/L [NaCl] = 800 mg/L pH = 2 T = 343.15 K	99.9%	[67]
Photocatalysis/PEDOT ^5^	Reaction Time = 60 min [MET]_0_ = 1 mg/L [PEDOT] = 0.5 g/L pH = 5.6 UV-B region	>99%	[68]
UV/sulfite process	Reaction Time = 30 min [MET]_0_ = 15 mg/L [Sulfite reagent] = 10 mM pH = 6	97%	[69]
Photocatalysis with La-doped tungsten trioxide (WO_3_) NPs ^6^	Reaction Time = 150 min [MET]_0_ = 200 mg/L [La-doped WO_3_] = 150 mg/L	93%	[70]

^1^ Electron beam irradiation, ^2^ vacuum ultraviolet, ^3^ peroxymonosulfate, ^4^ carbon nanotubes, ^5^ poly (3,4-ethylenedioxythiophene), ^6^ nanoparticles.

**Table 5:** Decomposition by-products generated from metformin degradation via advanced oxidation processes.

Advanced Oxidation Processes	Identified By-Products	Reference
TiO_2_-based photocatalysis	Ammonium (NH_4_^+^-N), nitrite (NO_2_^−^-N), nitrate (NO_3_^−^-N)	[110]
UV/sulfite	4,2,1-AIMT ^1^, methylbiguanide, metformin hydroperoxide, 2,4-AMT ^2^	[69]
Ozonation	NDMA ^3^, cyanamide	[111]
Advanced oxidation using sulfate radicals	N-cyanoguanidine, N,N-dimethyl-urea, N,N-dimethyl-cyanamide, N,N-dimethyl-formamide, glycolonitrile, guanidine	[61]
Photocatalysis with PEDOT	methylbiguanide, 4,2,1-AIMT	[68]
Photocatalysis and ozonation	Guanidine, aminoguanidine, biguanidine	[58]
Gamma radiolysis	Metformin hydroperoxide, methylbiguanide, 2,4-AMT, 4,2,1-AIMT	[112]
Heterogeneous photocatalytic oxidation—Fe_3_O_4_@rGO@ZnO/Ag-NPs catalyst	Guanidine, N,N-dimethylformimidamide, N-carbamimidoylformimidamide, 1,1-dimethylguanidine, N-(N,N-dimethylcarbamimidoyl)formimidamide	[66]
Photocatalysis—TiO_2_/ZrO_2_	Guanylurea, MBG ^4^, diMTF ^5^, MTFOOH ^6^, 4,2,1-AIMT	[55]
Oxy-cracking	NH_3_, monomethylamine (MMA) and dimethylamine (DMA), NDMA, DMF ^7^, DMU ^8^, DMG ^9^, hydroxyacetonitrile, guanylurea	[113]

^1^ 4-amino-2-imino-1-methyl-1,2-dihydro-1,3,5-triazine, ^2^ 2-amino-4-methylamino-1,3,5-triazine, ^3^ N-nitrosodimethylamine, ^4^ 1-methylbiguanide, ^5^ dimer of metformin, ^6^ hydroperoxide of metformin, ^7^ N,N-dimethylformamide, ^8^ N,N-dimethylurea, ^9^ dimethylguanidine.

## Data Availability

Data sharing is not applicable to this article.

## Abbreviations

The following abbreviations are used in this manuscript:

^•^OH	Hydroxyl radicals
2,4-AMT	2-amino-4 methylamino-1,3,5-triazine
4,2,1-AIMT	4-amino-2-imino-1-mehtyl-1,2-dihydro-1,3,5-triazine
AFR	Africa
AOPs	Advanced oxidation processes
BTEX	Benzene, toluene, ethylbenzene and xylenes
BUF	Buformin
CB	Conduction band
CNT	Carbon nanotubes
diMTF	Dimer of metformin
DM	Diabetes mellitus
DMA	Dimethylamine
DMF	N,N-dimethylformamide
DMG	Dimethylguanidine
DMU	N,N-dimethylurea
DMT1	Diabetes mellitus type 1
DPP-4	Dipeptidyl peptidase
EBI	Electron beam irradiation
ECs	Emerging contaminants
EU	European Union
EUR	Europe
FGZAg	Fe_3_O_4_@rGO@ZnO/Ag-NPs
GLP-1	Glucagon-like peptide-1
H_2_O_2_	Hydrogen peroxide
IDF	International Diabetes Federation
Kow	Octanol–water partition coefficient
LDL	Low-density lipoprotein
MBG	1-mehtylbiguanide
MENA	Middle East and North Africa
MET	Metformin
MMA	Monomethylamine
MODY	Maturity Onset Diabetes of the Young
MTFOOH	Hydroperoxide of metformin
NAC	North America and Caribbean
NDMA	N-nitrosodimethylamine
NPs	Nanoparticles
PEDOT	Poly (3,4-ethylenedioxythiophene)
PHF	Phenformin
PMS	Peroxymonosulfate
rGO	Reduced graphene oxide
SCA	South and Central America
SEA	South-east Asia
SGLT2	Sodium–glucose co-transporter 2
t-BA	Tert-butyl alcohol
TiO_2_	Titanium dioxide
UV	Ultraviolet
VB	Valence band
VUV	Vacuum ultraviolet
WO_3_	Tungsten trioxide
WP	Western pacific
WWTPs	Wastewater treatment plants
ZrO_2_	Zirconium dioxide
